# Personality Profile of Parents of Children with Attention Deficit Hyperactivity Disorder

**DOI:** 10.1155/2014/212614

**Published:** 2014-11-26

**Authors:** Hossein Dadashzadeh, Shahrokh Amiri, Ahmad Atapour, Salman Abdi, Mahan Asadian

**Affiliations:** ^1^Clinical Psychiatry Research Center (CPRC), Tabriz University of Medical Sciences, Tabriz, Iran; ^2^Department of Psychiatry, Razi Mental Hospital, El Goli Boulevard, Tabriz, East Azerbaijan 51677, Iran; ^3^Tabriz University of Medical Sciences, Tabriz, Iran; ^4^Tehran University of Medical Sciences, Iran

## Abstract

*Objectives.* The present study was carried out aiming to identify the personality profile of parents of children with Attention Deficit Hyperactivity Disorder (ADHD). *Methods.* This study is of a descriptive, analytic, cross-sectional type in which parents of 6–12-year-old children with ADHD who were referred to the Bozorgmehr Psychiatric Clinic, affiliated with Tabriz University of Medical Sciences, were enrolled. ADHD was diagnosed according to the criteria of DSM-IV-TR and a quasi-structured diagnostic interview (K-SADS-PL). The personality profile of the parents was assessed with the Millon Clinical Multiaxial Inventory-III (MCMI-III). *Results.* According to the findings of this study, the most common personality problems based on the assessment scales in the MCMI-III belonged to the clinical patterns of depressive personality in 43 persons (25.3%), histrionic personality in 34 persons (20%), and compulsive personality in 29 persons (17.1%). According to discriminant analysis, four scales of somatoform, sadistic, dependence, and though disorder were direct and antisocial scale was reverse significant predictors of membership in the women group. *Conclusion.* According to the findings of this pilot study, personality disorders are prevalent in parents of ADHD children and mothers suffer from personality disorders more than fathers.

## 1. Introduction

According to the Diagnostic and Statistical Manual of Mental Disorders, Attention Deficit Hyperactivity Disorder (ADHD) is one of the most common psychiatric diagnoses in children and adolescents and affects individual, social, and family aspects of the person [1]. The prevalence of ADHD in elementary schools in Tabriz, at north-west of Iran, has been reported as 9.7% [2]. ADHD is also seen in comorbidity with many abnormalities; for example, the prevalence of comorbidity of psychiatric disorders with ADHD children has been reported as 62.5% [3]. Since the exact cause for ADHD has not been found, researchers focus on the psychopathology of ADHD and the family environment of ADHD children from different aspects [4].

According to systemic view to family as an explanatory and therapeutic approach to psychiatric disorder, disturbed behavior is created within a family system; thus the affected individual should be treated in the family with the involved persons [5]. Therefore, a comprehensive view on the aspects of mental damage of parents is a practical requirement for clinicians to identify the treatment direction of parents and ADHD children. In this regard, studies in Iran also show that the lifetime prevalence of depression in mothers and fathers of ADHD children is 48.1% and 43%, respectively [6]. Another study showed that 87% of fathers of ADHD children had a history of substance abuse, and fathers suffered from psychiatric illness with a higher prevalence than mothers [7].

Other researches on the problems of parents of ADHD children show that the parents of ADHD children showed higher levels of ADHD symptoms, depression, and depressive personality disorder compared to the parents of normal children, and in a more accurate orientation, the study showed that mothers were more depressed and fathers had alcohol-related problems [8]. Moreover, in terms of personality problems, the parents of ADHD children were suffering from more antisocial and hystrionic personality disorders compared with the parents of non-ADHD children [9].

Parents' mental health decrement gains more importance because it increases the risk of psychological problems in children. In this regard, a previous report suggested that parents with psychiatric problems have children who suffer from a diverse range of psychiatric disorders. If both parents have a history of psychiatric disorder, the risk of severe psychiatric disorders in children may be increased 13 times [10]. Accordingly, since ADHD is a common problem and has an enormous psychological burden for families, more comprehensive and holistic interventions are required in family aspects (parents and other children) to achieve more effective therapeutic interventions [11].

Given that the parents' personality reflects their general mental status and has a persistent and penetrating internal pattern of behavior and experience [1], personality disorders of parents can predispose their children to other psychiatric disorders [12]. Identifying personality disorders of parents helps better understand and predict their future behaviors and fundamental factors of ADHD children problems. Scientific necessity and few studies on personality profiles of parents of ADHD children were the main reasons for conducting this research.

## 2. Materials and Methods

This descriptive, analytic, cross-sectional study was performed in 2013. The subjects were selected through convenience method from parents of 6- to 12-year-old children with ADHD referred to Bozorgmehr Psychiatric Clinic (affiliated with Tabriz University of Medical Sciences).

### 2.1. Participants

One hundred and seventy parents of ADHD children participated in the study; 97 of them (57.5%) were women and 73 (42.95%) were men. The mean age of the parents was 35.46 ± 6.78 years with an age range of 22–53 years.

### 2.2. Inclusion and Exclusion Criteria

The inclusion criteria were informed consent to participate in the study, primary diagnosis of ADHD in children, age range of 6–12 years for ADHD children, diagnosis of ADHD according to DSM-IV-TR, and literacy of parents with at least 8th grade. Children or parents are excluded from the study in case of having any severe physical/mental disabilities.

## 3. Instruments

### 3.1. Millon Clinical Multiaxial Inventory-III (MCMI-III)

The third version of MCMI consists of 175 yes or no questions and is performed on people over 18 years. MCMI-III is one of the most important tools for objective assessment of axis I clinical symptoms of and axis II personality disorders according to DSM-IV. MCMI-III consists of 24 clinical scales that are classified in four categories.

(a) The eleven personality clinical scales are as follows: schizoid, avoidant, depressive, dependent, histrionic, narcissistic, antisocial, sadistic, compulsive, negativistic, and masochistic scales; (b) three severe personality pathology scales are as follows: schizotypal, borderline, and paranoid scales; (c) seven clinical syndrome scales are as follows: anxiety, somatoform, bipolar, dysthymia, alcohol dependence, drug dependence, and posttraumatic stress disorder scales; (d) three severe clinical syndrome scales are as follows: thought disorder, major depression, and delusional disorder scales.

Each item is scored between 1 and 2, according to the symptoms of each clinical scale. Persian version has an acceptable validity and reliability like the original version. An Iranian study reported the validity of the Persian version of MCMI-III between 0.58 and 0.83 [13].

Filling of the questionnaire takes 20–30 minutes and the respondent must have at least the 8th grade of education and age of 18 years. This questionnaire was used in this study to identify the personality profiles of parents of ADHD children.

### 3.2. Data Analysis

All statistical analyses were performed with SPSS-17. Descriptive statistical methods were used to determine the prevalence (frequency and percentage) of personality disorders. The frequency of personality disorder and multiplicity of personality disorder were compared between mothers and fathers with Fisher's exact test and chi-square. The independent *t*-test was used to compare the mean scores of MCMI-III in mothers and fathers of children with ADHD. The discriminant analysis was used to predict the group membership according to MCMI-III scores. Power values less than 0.05 were considered significant.

## 4. Results

The mean age of children was 8.09 ± 2.22 years and 63 patients (21.2%) were females and 134 (78.8%) were male.

Eighty children had comorbidities and 90 patients had not. The prevalence of psychiatric disorders comorbidities in children with ADHD was 46.5% for oppositional-defiant disorder, 3.5% for enuresis, 2.9% for motor tic, social phobia, and generalized anxiety disorder each, 2.4% for obsessive-compulsive disorder, and 0.6% for Tourette's syndrome.

In terms of parents' education, 61 persons (35%) had school education, 75 persons (44.1%) diploma, 11 persons (6.5%) associate degree, 19 persons (11.2%) B.S. degree, and 4 patients (2.4%) M.S. and Ph.D. degrees.

Based on the results in Table 1, in the clinical personality scale, the most prevalent disorders were depressive disorder in 43 patients (25.3%), histrionic disorder in 34 patients (20%), and compulsive disorder in 29 patients (17.1%), while the lowest prevalence of personality disorders in this scale was sadistic (1.2%) and antisocial and narcissistic (2.4%).

In the severe personality pathology scale, the prevalence of disorders was 5.3% for schizotypal, 3.5% for borderline, and 2.9% for paranoid scales.

In the clinical symptoms scale, the most prevalent disorders were dysthymic (8.8%) and anxiety, mania, and PTSD (4.7%), while the lowest prevalent disorders were alcohol dependence (0.6%) and drug dependence (1.2%).

In the severe clinical syndrome scale, the prevalence of disorders was 4.1% for major depression, 2.4% for thought disorder, and 1.8% for delusional disorder.

Based on Fisher's exact test, the total prevalence of personality disorders in 24 scales of MCMI-III was 95 persons (55.9%) with 62 women (63.9%) and 33 men (45.2%) so that personality disorders frequency was higher in women (*P* < 0.05, *χ* ^2^ = 5.91). According to the results of chi-square test, women suffered more than men from multiplicity of personality disorders so that 40 men (57.8%) and 35 women (36.1%) were free of personality disorders; 18 men (24.7%) and 27 women (27.8%) had only one personality disorder; and 15 men (20.5%) and 35 women (36.1%) had more than one personality disorder (*P* < 0.05, df = 2, *χ* ^2^ = 6.88).

Based on Fisher's exact test, 49 children (61.3%) without comorbidities and 46 children (51.1%) with comorbidities had parents with personality disorder. In addition, 31 children (38.8%) without comorbidities and 44 children (48.9%) with comorbidities had parents with no personality disorder so that the frequency of personality disorders in parents of ADHD children with and without comorbidities was similar (*P* < 0.21, *χ* ^2^ = 1.76).

To compare the incompatible personality profiles of patients in the studied groups, the scores for each item of the MCMI-III subscales were summed and then the mean raw scores of the studied groups were compared using the independent *t*-test (Table 1). The results of *t*-test showed that the groups of women and men differed in the mean of symptoms of 12 indices of incompatible personality (*P* < 0.05), so that women had more symptoms of avoidant, depressive, dependent, negativistic, borderline, anxiety, somatoform, dysthymia, posttraumatic stress disorder, thought disorder, and major depression (*P* < 0.05). In addition, men had higher mean scores of drug dependence (*P* < 0.05). No difference existed between men and women in total scores of personality clinical scales (*P* > 0.05); however, the mean scores of severe personality pathology scales, clinical syndrome scales, and severe clinical syndrome scales were higher in women than men with 95% confidence.

In order to differentiate men and women based on the mean scores of the MCMI-III scales, the discriminant analysis statistical method was used through stepwise procedure with entrance of 24 scales of MCMI-III. The results of discriminant analysis for separation of women and men showed that the five scales of somatoform, antisocial, sadistic, thought disorder, and dependent were discriminators of male and female parents, respectively, in five steps, so that somatoform, sadistic, dependent, and thought disorders directly and antisocial disorder inversely predicted membership in the women group. According to the mentioned five incompatible personality profiles, 68 women (70.1%) out of 97 and 59 men (80.8%) out of 73 were correctly diagnosed and had personality problem. In total, averagely 74.7% of the plotted profile had the ability to predict males and females (Table 1).

## 5. Discussion 

The present study aimed to determine the personality profiles of parents of 6–12-year-old children with ADHD in Tabriz and found that the total prevalence of personality disorders based on MCMI-III was 55.9%. The highest prevalence of personality disorders was 25.3% for depressive, followed by 20% for histrionic, and 17.1% for compulsive personality clinical patterns.

Consistent with these findings, a study reported that 52% of parents of ADHD children suffer from a psychiatric disease [7]. On the other hand, the total prevalence of personality disorders in general population has been reported as 10.6% [14].

The prevalence in inpatient and outpatient samples of psychiatric services has been reported 17.6% and 10.3%, respectively [15]. Therefore, it seems that the pattern of personality disorders was more prevalent in parents of children with ADHD [8, 9].

Another part of findings of this study showed that the prevalence of personality disorders in parents of ADHD children with and without comorbidities was similar. But all MCMI-III personality disorders were more in mothers than fathers (63.9% versus 45.2%) and mothers suffered from higher intensity (higher mean scores and more frequent comorbidities of personality disorder) so that somatoform, sadistic, dependent, and thought disorders were direct predictors of mothers of ADHD children and antisocial disorder was predictor of fathers of ADHD children.

A study showed that mothers of children with ADHD+ODD/CD suffered from mood disorders, anxiety disorders, and dependence on stimulant/cocaine and fathers suffered from alcohol consumption [16]. Another report indicated that symptoms of anxiety and depression in mothers of children with ADHD were more prevalent [17]. In another study, it was reported that fathers suffered from psychiatric illness with higher frequency than mothers so that 87% of parents of ADHD children had a history of substance use disorder [7]. It seems that the main causes of difference in the findings of studies which are less consistent are different statistical population, sample sizes, research tools, and orientation in identifying the difficulties of parents so that the small sample size of 50 parents in the study of Latha et al. led to reduced accuracy of the findings [7], while Segenreich et al. used nonclinical samples and the State-Trait Anxiety Inventory and Beck Depression Inventory to measure anxiety and depression [17] and Margari et al. examined the psychological aspects of damage [8].

In total, according to the findings of this study, parents of ADHD children had high prevalence of personality disorders, although high prevalence of personality disorders cannot be considered as a consequence or cause of ADHD; rather, these findings indicate high levels of damage in parents of ADHD children, reminding us of the importance of clinical interventions and psychiatric study and treatment of parents of ADHD children.

Since no matched control group existed in this study, its comparison with previous studies may be less accurate; however, our findings in a general population sample showed a great difference in personality disorders such as depression, histrionic and compulsive disorders, emphasizing higher prevalence of personality disorders in parents of children with ADHD.

But the affection of children from their parents which can facilitate children's behavioral problems or exacerbate children's problems is more important than the prevalence of personality problems in parents; it was reported that attention deficit and associated disorders of children and depression of mothers were significantly correlated with reduced maternal emotional care; also, hyperactivity and impulsivity of children and neurotic traits of mothers were significantly associated with overprotective mothers and correlated with impaired mother-child interaction and poor support family [18].

Our findings also confirmed the view of family system and emphasized that children with psychiatric disorders had parents who suffered from psychiatric disorders [10, 19, 20]. According to the theory of family system, the behavior of each family member (especially parents) affects the behavior of other family members (especially children) and, thus, presence of any psychiatric disorder in parents affects children behavior and their behavioral problems [21]. However, it should not be ignored that the genetic similarity of parents with children can enhance their susceptibility to psychiatric disorders.

However, the approach of problem reduction emphasizes that psychological interventions combined with therapeutic interventions for children with ADHD will help comprehensive treatment for coping with the fundamental possible elements in children with ADHD so that previous studies indicated that family intervention and parent-child interaction therapy are effective in reducing ADHD problems [11, 22].

Despite the limitations of a study, its findings could be applicable. Accordingly, lack of a control group to compare the prevalence of personality disorders in parents was a limitation for this study. This limitation indicates the need for further study and understanding other aspects of psychological damage and interpersonal relationships of family members of children with ADHD.

## 6. Conclusion

The prevalence of personality disorders in parents of ADHD children was 55.9% according to MCMI-III. Personality disorders were more prevalent in mothers than fathers of children with ADHD (63.9% versus 45.2%). The prevalence of somatoform, sadistic, dependent, and thought disorders was higher in mothers while antisocial disorder was higher in fathers of children with ADHD. Considering the results of this study, treatment of personality problems of parents of ADHD children is a helpful goal for psychiatric interventions along with therapeutic interventions for ADHD children.

## Figures and Tables

**Table 1:** Prevalence of personality disorders of parents of ADHD and *t*-test result of personality comparison between male and female.

MCMI-III scales	*N* (%)	Female *N* (%)	Male *N* (%)	Female mean (SD)	Male mean (SD)	*P*-value^a^
Personality clinical scales	93 (54.7)	61 (62.9)	32 (43.8)	104.85 (33.75)	94.17 (38.93)	1.91
Schizoid	6 (3.5)	2 (2.1)	4 (5.5)	8.64 (4.17)	7.58 (4.76)	1.54
Avoidant	10 (5.9)	8 (5.2)	2 (2.7)	7.38 (4.76)	5.57 (4.24)	2.56^*^
Depressive	43 (25.3)	33 (34)	10 (13.7)	9.23 (6.23)	6.09 (5.49)	3.40^**^
Dependent	11 (5.6)	9 (9.3)	2 (2.7)	9.65 (5.07)	7.34 (4.05)	3.20^**^
Histrionic	34 (20)	21 (21.6)	13 (17.8)	12.11 (3.23)	12.69 (3.18)	1.17
Narcissistic	4 (2.4)	2 (2.1)	2 (2.7)	10.90 (3.81)	12.12 (4.22)	1.95
Antisocial	4 (2.4)	0	4 (5.5)	5.41 (3.40)	6.38 (4.73)	1.55
Sadistic	2 (1.2)	0	2 (2.7)	9.51 (4.34)	8.13 (5.25)	1.87
Compulsive	29 (17.1)	18 (18.6)	11 (15.1)	14.69 (3.61)	14.83 (3.77)	0.23
Negativistic	21 (12.4)	17 (17.5)	4 (5.5)	10.01 (5.94)	7.67 (5.80)	2.57^*^
Masochistic	5 (2.9)	1 (1)	4 (5.5)	7 (4.43)	5.72 (4.99)	1.75
Severe personality pathology scales	12 (7.1)	8 (8.2)	4 (5.5)	21.25 (13.10)	16.93 (14.01)	2.06^*^
Schizotypal	9 (5.3)	5 (5.2)	4 (5.5)	5.68 (4.80)	4.34 (5.31)	1.71
Borderline	6 (3.5)	5 (5.2)	1 (1.4)	7.22 (5.57)	4.91 (4.91)	2.81^**^
Paranoid	5 (2.9)	2 (2.1)	3 (4.1)	8.35 (4.49)	7.67 (4.93)	0.93
Clinical syndrome scales	23 (13.5)	15 (15.5)	8 (11)	33.16 (22.46)	24.30 (23.68)	2.48^*^
Anxiety	8 (4.7)	6 (6.2)	2 (2.7)	5.35 (4.28)	3.10 (3.96)	3.48^**^
Somatoform	6 (3.5)	4 (4.1)	2 (2.7)	5.85 (4.24)	3.41 (3.85)	3.86^**^
Bipolar	8 (4.7)	4 (4.1)	4 (5.5)	5.12 (4.26)	4.15 (4.08)	1.50
Dysthymia	15 (8.8)	11 (11.3)	4 (5.5)	5.93 (5.18)	3.72 (4.62)	2.88^**^
Alcohol dependence	1 (0.6)	0	1 (1.4)	3.58 (2.08)	3.52 (2.64)	0.18
Drug dependence	2 (1.2)	0	2 (2.7)	2.64 (2.19)	3.58 (3.58)	2.10^*^
Posttraumatic stress disorder	8 (4.7)	7 (7.2)	1 (1.4)	4.65 (4.95)	2.79 (4.10)	2.61^*^
Severe clinical syndrome scales	10 (5.9)	7 (7.2)	3 (4.1)	17.42 (11.87)	12.54 (12.15)	2.62^**^
Thought disorder	4 (2.4)	2 (2.1)	2 (2.7)	6.72 (5.02)	4.69 (5.04)	2.59^**^
Major depression	7 (4.1)	5 (5.2)	2 (2.7)	6.87 (5.45)	4.06 (4.73)	3.51^**^
Delusional disorder	3 (1.8)	1 (1)	2 (2.7)	3.82 (2.74)	3.78 (3.16)	0.09

^a^df = 168, ^*^ *P<0.01* and ^**^ *P<0.001*.
